# A Less Common Anatomical Variant of Bilateral Multiple Lesser Metatarsal Sesamoids With Radiologic and Clinical Correlation

**DOI:** 10.7759/cureus.84771

**Published:** 2025-05-25

**Authors:** Christos Lyrtzis, Dimitra Mpei, Kallisti St John, George Paraskevas, Nikolaos Lazaridis

**Affiliations:** 1 Department of Anatomy and Surgical Anatomy, Faculty of Health Sciences, Medical School, Aristotle University of Thessaloniki, Thessaloniki, GRC; 2 School of Medicine, Aristotle University of Thessaloniki, Thessaloniki, GRC

**Keywords:** accessory bones, metatarsalgia, orthopedic case study, radiology, sesamoid bones

## Abstract

Accessory or supernumerary bones are bones that are not normally present in the body but can be found as an anatomical variant. While hallucal sesamoids are common, lesser metatarsal sesamoids are uncommon and often underreported. These ossicles develop through endochondral ossification and aid biomechanics by enhancing muscle function and reducing tendon friction. This report presents the first documented case of bilateral multiple lesser metatarsal sesamoids involving several metatarsophalangeal joints, supported by radiologic and clinical correlation.

A 48-year-old woman presented with bilateral plantar foot pain, exacerbated by weight-bearing activities. Radiographs and computed tomography (CT) confirmed multiple lesser metatarsal sesamoids bilaterally, along with ossa peronea. Conservative management, including non-steroidal anti-inflammatory drugs, footwear modifications, and physical therapy, led to symptom improvement after six months.

Lesser metatarsal sesamoids are usually asymptomatic but can contribute to metatarsalgia. Recognizing these ossicles as anatomical variants is essential to avoid misdiagnosis and unnecessary interventions. This case underscores their potential role in forefoot pain and diagnostic challenges.

## Introduction

Accessory bones are additional ossicles not typically found in the human skeleton. Numerous accessory ossicles can be found in the foot and recognizing them is essential to avoid misdiagnosing fractures and to identify sources of symptoms. Accessory ossicles and sesamoid bones can be found in the ankle and foot alongside other major anatomical variations, such as bipartitions and coalitions [1].

Accessory intermetatarsal bones are extra bones found between the metatarsals. Sesamoid bones in the foot are categorized as hallucal, interphalangeal's joint sesamoids and lesser metatarsal sesamoids [2]. Embedded within tendons, sesamoids enhance muscle function and reduce friction. While sesamoids of the first metatarsophalangeal joint or hallucal sesamoids are present in all individuals, lesser metatarsal sesamoids are rare and relatively underreported. In fact, they are considered to be accessory bones when encountered [3].

A common pitfall is mistaking bipartite hallucal or lesser metatarsal sesamoids for a fractured sesamoid. Other sesamoid-related pathologies include degenerative changes, infections and osteonecrosis [2]. This case report presents a rare instance of a symptomatic patient with bilateral multiple lesser metatarsal sesamoids, showcasing their anatomical features, associated pathology and diagnostic challenges.

## Case presentation

A 48-year-old woman presented to our clinic presenting with bilateral plantar foot pain, more pronounced on the right side. She reported pain during weight-bearing activities, along with swelling and tenderness beneath the metatarsal heads. Physical examination revealed metatarsalgia with plantar callosities but no hallux valgus or other lesser toe deformities.

**Figure 1:**
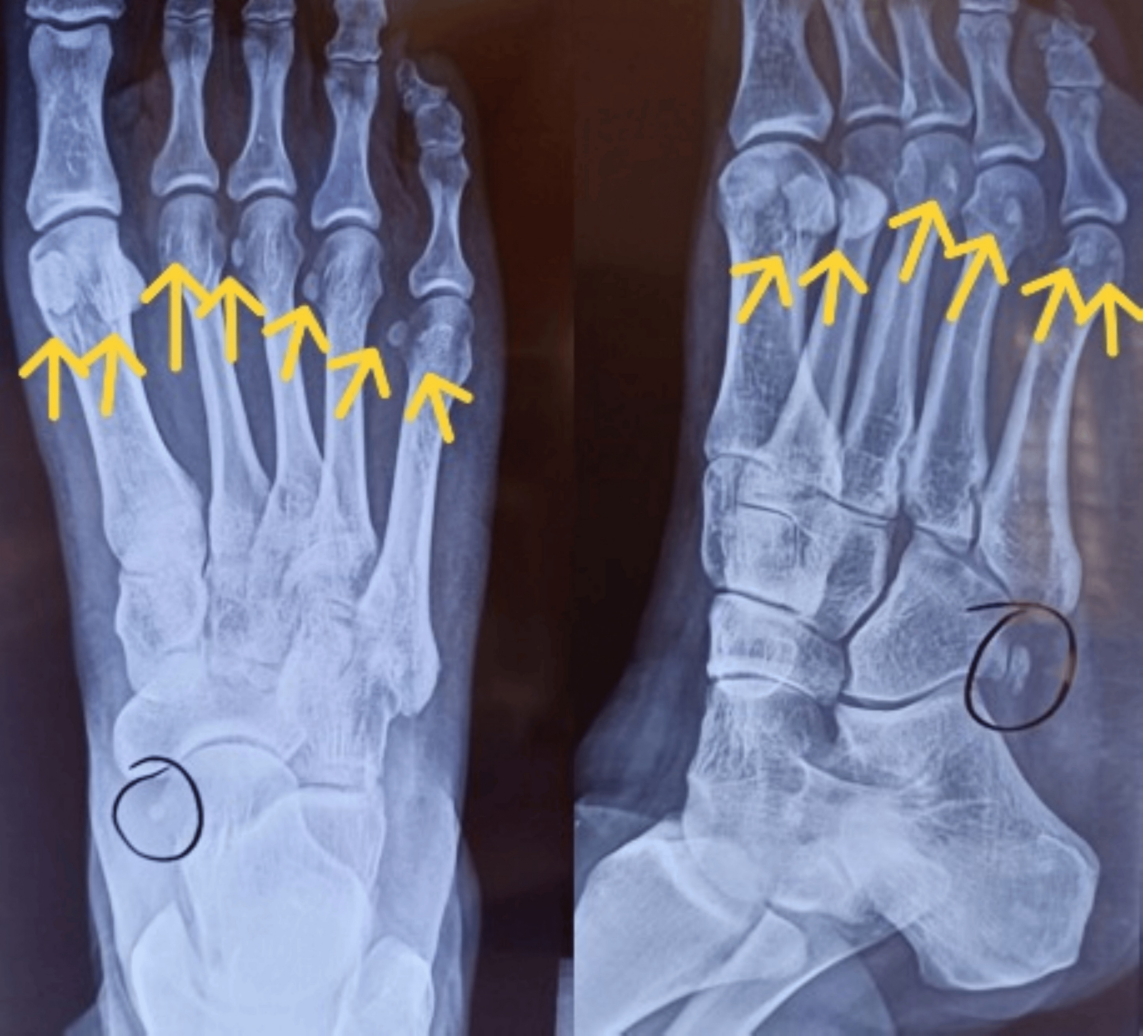
Weight-bearing dorsoplantar and non-weight-bearing oblique radiographs of the right foot

Weight-bearing dorsoplantar and non-weight-bearing oblique radiographs of the right foot revealed the presence of sesamoid bones in the lesser metatarsophalangeal joints (Figure 1). Additionally, multiple ossa peronea were found bilaterally.

**Figure 2:**
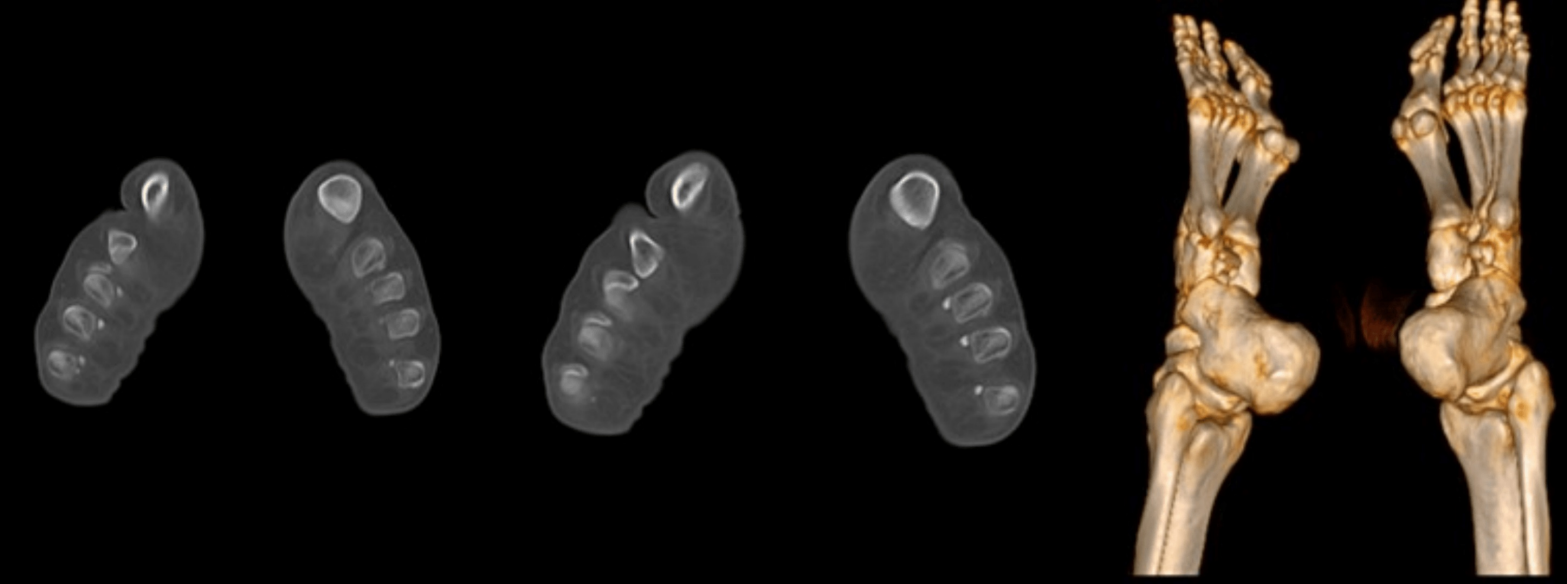
Computer tomography and 3D reconstruction of the feet

The clinical team then decided to perform computer tomography (CT) of both feet. CT revealed on the right foot two ossicles under the first and fifth metatarsal heads, and single ossicles located under the second, third, and fourth metatarsal heads. Also, on the left foot were found two ossicles under the fourth and first metatarsal heads, and single ossicles under the third and fifth metatarsal heads. The sesamoids ranged from 3.4 to 8.0 mm in length. (Figure 2).

Conservative treatment of the patient was initiated, including weight loss, non-steroid anti-inflammatory drugs (Naproxen 500 mg twice daily for 20 days) to reduce inflammation and pain, cryotherapy, rest, shock-absorbing and arch-supporting insoles and footwear modifications to reduce discomfort. Physical therapy was also included in the treatment plan, involving stretching and strengthening exercises. At the six-month follow-up, the patient’s metatarsalgia had improved.

## Discussion

Lesser metatarsal sesamoids, formed through endochondral ossification, are influenced by developmental and biomechanical factors. Variability in their ossification leads to differences in number and location [4]. They enhance muscle force production, reduce tendon friction, and have an impact on joint biomechanics [5].

Prevalence varies across populations. Lesser metatarsal sesamoids are typically found at the second and fifth metatarsophalangeal joints, with occasional presence at the third and fourth, and some studies suggest a prevalence of up to 5% [1,6]. Research on Turkish individuals reported sesamoid presence in 2.8%, 0.5%, 1.0%, and 15.1% at the second, third, fourth, and fifth metatarsophalangeal joints, respectively [6]. Another Turkish study of 984 patients found sesamoid bones in 0.4%, 0.2%, 0.1%, and 4.3% of the cases at these joints [7]. In a study of 505 Italian women with hallux valgus, prevalence was 15, six, and nine cases at the second, third, and fourth metatarsophalangeal joints, respectively [8]. One reported case involved a 68-year-old woman with lesser metatarsal sesamoids in all metatarsophalangeal joints [3].

Lesser metatarsal sesamoids can affect load distribution, biomechanics, and joint stability during gait. Their size and position influence pressure distribution under the metatarsal heads. The presence of these ossicles can complicate the diagnosis and treatment of other foot pathologies. They are commonly located within the tendons of the interosseous muscles at the plantar aspect of the lesser metatarsal heads, with varying morphology from small, round ossicles to elongated structures.

Diagnosis of accessory intermetatarsal bones is primarily through imaging. Plain radiographs are typically the first imaging modality used to identify these bones. Multiple views, including oblique and weight-bearing views, are essential for accurate visualization [5,9]. Advanced imaging techniques, such as CT, aid in providing a more detailed assessment, especially if there is suspicion of associated soft tissue pathology or other foot abnormalities. Further, ultrasonography or nuclear imaging may help in challenging cases as well as evaluating foot pain secondary to accessory ossicles. MRI is useful for diagnosis as it shows abnormalities in the sesamoids and surrounding soft tissue inflammation, and bone scintigraphy may show increased uptake in the sesamoids [1]. They appear as small, round, or oval ossicles between the metatarsal bones.

Accessory sesamoids are usually asymptomatic and are incidentally identified on radiographs. Sometimes they can cause pain, particularly after repetitive stress or trauma [10]. Sesamoiditis is a painful inflammatory condition of the sesamoid bones and surrounding tissues. The pain is exacerbated by walking, particularly when wearing certain flexible thin-soled or high-heeled shoes, and the area may be warm and swollen [11]. Fractures of the lesser metatarsal sesamoids, though rare, can occur from direct trauma or repetitive loading. Symptomatic sesamoids may contribute to metatarsalgia, as seen in our case, especially if enlarged or malpositioned. Additionally, their presence can complicate the diagnosis and treatment of other pathologies in this region. Misdiagnosing them as fractures or pathological conditions may lead to unnecessary interventions.

Treatment is normally conservative, including rest, orthotics, non-steroidal anti-inflammatory drugs, physical therapy, weight loss, cryotherapy, rest, and incorporating footwear modifications like supportive insoles. Stretching and strengthening exercises can improve foot mechanics and reduce stress on accessory bones. Surgical intervention is very rare and reserved for cases of persistent pain or impingement [11].

It is already known that lesser metatarsal sesamoid bones are rare anatomical variants found at the metatarsophalangeal joints. Their presence can influence foot biomechanics, load distribution, and joint stability [5]. They are often incidental findings on imaging but may be mistaken for fractures or associated with pain syndromes like metatarsalgia [10]. Diagnosis relies primarily on radiographic evaluation, though advanced imaging may be required in symptomatic cases. Treatment is typically conservative, focusing on pain management and biomechanical correction [11].

This study presents a rare case of bilateral multiple lesser metatarsal sesamoids, highlighting their anatomical distribution, potential for misdiagnosis, and clinical significance. It reinforces the need for careful radiographic assessment to distinguish these accessory bones from pathological conditions. This is the first reported case of a patient with a plethora of lesser metatarsal sesamoids bilaterally, expanding current knowledge on their variability and impact on foot pathology.

## Conclusions

Recognizing sesamoids and accessory ossicles as normal anatomical structures prevents unnecessary interventions; however, their role in painful foot pathologies should not be overlooked. Lesser metatarsal sesamoids significantly influence forefoot biomechanics and can potentially cause other symptoms. While multiple accessory lesser metatarsal sesamoids have been reported in literature, this is the first case documenting their bilateral presence at multiple metatarsophalangeal joints. This case underscores the anatomical variations, diagnostic challenges, and clinical implications of these rare ossicles. Future research or outlining diagnostic protocols for similar cases can link the presence of multiple lesser metatarsal sesamoids to symptoms.
